# The effects of ozone and melatonin on busulfan-induced testicular damage in mice

**DOI:** 10.5935/1518-0557.20200081

**Published:** 2021

**Authors:** Mahin Taheri Moghadam, Reza Dadfar, Layasadat Khorsandi

**Affiliations:** 1 Cellular and Molecular Research center, Ahvaz Jundishapur University of Medical Sciences, Ahvaz, Iran; 2 Department of Anatomical Sciences, Faculty of Medicine, Ahvaz Jundishapur University of Medical Sciences, Ahvaz, Iran

**Keywords:** Busulfan, melatonin, mice, ozone, stress oxidative, testis

## Abstract

**Objective:**

Busulfan is one of the most common chemotherapeutic drugs and has the ability to induce apoptosis in testicular germ cells, which leads to infertility. In this study, the effects of ozone therapy and melatonin were evaluated on testicular disorders induced by busulfan.

**Methods:**

In this study, we divided 24 male mice into four groups: control group, groups treated with busulfan, busulfan/melatonin, and busulfan/ozone. At the end of a 35-day period, blood samples were taken from the mice and their testosterone levels were measured. Both of the mice’s testes were removed and weighed, afterwards, each one of them was used for evaluation of morphology by Johnson’s score, as well as for measuring the diameter and thickness of seminiferous tubules. The other testis was homogenized for measuring Malondialdehyde (MDA) and antioxidant status using Catalase (CAT), Super Oxide Dismutase (SOD), and Total Antioxidant Capacity (TAC) levels. Epididymis spermatozoa were also used to evaluate motility, morphology, and sperm count.

**Results:**

Busulfan significantly reduced the testis quality (weight, sperm parameters, testosterone, CAT, SOD, and TAC levels) and increased MDA and destruction of seminiferous tubules compared to the control group. Ozone and melatonin treatments significantly increased testis quality, sperm parameters, MDA, and antioxidant status, but they did not affect the TAC level.

**Conclusions:**

This study showed that similar to melatonin, ozone can reduce the effect of busulfan toxicity on mice testis. However, further studies are needed to understand the precise mechanism of ozone function on testis.

## INTRODUCTION

Busulfan is one of the most commonly used chemotherapy drugs. It is used before, after bone marrow transplantation, and for other cancers as well (Nieto *et al*., 2012). However, busulfan damages the normal cells too. It induces reactive oxygen species (ROS) production, apoptosis, and damage to cell DNA (Probin *et al*., 2007). ROS is one of the most important factors involved since it has a major impact in the development of oxidative stress (Probin *et al*., 2007). It also has the ability to induce apoptosis and kill the testicular germ cells, and disrupt spermatogenesis that in many cases, leads to infertility (Choi *et al*., 2004). It seems that busulfan inhibits the spermatogenesis process, especially by oxidative damage and its alkylating properties (Jansz & Pomerantz, 1985). It has been used in studies concerning the testes of different species for inducing azoospermia prior to transplantation of germinal cells to the testes (Brinster & Avarbock, 1994; Brinster & Nagano, 1998).

Today, research has shown that using antioxidants, including vitamin E and melatonin, leads to a renewed spermatogenesis process in patients treated with busulfan (Mohammad Ghasemi et al., 2010a; 2010b; Rohnavaz *et al*., 2016). Melatonin, one of the epiphysis gland secretions, is effective in regulating some physiological phenomena. It has several functions such as being a neurotransmitter and a reproductive regulator, and it adjusts the immune system and body temperature. The role of melatonin has been noted in recent years as a highly effective and powerful antioxidant and anti-free radical substance. It has been shown that melatonin could easily pass through the cell membrane and protect DNA and cells against harmful factors like oxidative stress, inflammation, apoptosis, cancer, and aging (Sanchez-Hidalgo *et al*., 2009; Hussein *et al*., 2006). Melatonin receptors have been identified on spermatozoa, which play a large role in reducing the apoptosis induced by H_2_O_2_ (Radogna *et al*., 2008) and oxidative stress (Ozen *et al*., 2008). These studies have shown that melatonin may play a protective role in the membrane’s lipid structure and affect the performance of mitochondria in spermatozoa and consequently, sperm motility (Rojansky *et al*., 1992). Another study showed that melatonin inhibited the apoptosis and oxidative stress of mice Leydig cells through a silent information regulator 1 (SIRT1) factor-dependent mechanism (Xu *et al*., 2019). This factor activates forkhead box O1 to synthesize SOD and CAT, resulting in increased cellular resistance to oxidative stress (Brunet *et al*., 2004). Moreover, melatonin acts as a local modulator of the endocrine activity in Leydig cells and influences cellular proliferation and energy metabolism in Sertoli cells, and can consequently regulate steroidogenesis. These studies suggest that melatonin is a key factor in steroidogenesis regulation (Yu *et al*., 2018).

In addition, ozone has been investigated as a therapeutic agent for treating different ROS-mediated physiopathological events (Bocci, 2004; Peralta *et al*., 2000). It is a molecule composed of three oxygen atoms (O_3_), which is due to the unstable dynamics of the mesomorphic state. Its half-life is 40 minutes at 20ºC and 140 minutes at 0ºC. Although O_3_ has harmful effects, researchers believe that they have many therapeutic effects (Di Paolo *et al*., 2004, Bocci, 1996). Ozone therapy, applied as a gas mixture of oxygen and ozone, has been widely used in a number of diseases such as chronic cutaneous ulcers, peritonitis, infected wounds, ischemic diseases, and joint problems (Bocci, 2011). It acts as an effective oxidative stress regulator, mainly by stimulating the antioxidant system (Sagai & Bocci, 2011). One study showed that ozone administration in diabetes mellitus rats reduced the levels of oxidative stress markers and improved renal antioxidant enzyme activities, as well as SOD, CAT, and GPx activities, especially when rats were treated with a combination of ozone and insulin (Morsy *et al*., 2010). In addition, some studies have concluded that it can provide a new tool to protect organs from ischemia reperfusion injury (IRI) (Chen *et al*., 2008). Results of previous studies have shown that ozone therapy, via improving the immune system, significantly protects testicular function in the setting of testicular torsion/ischemia, it provides protection against the effects of gonadotoxic agents, and treats bacterial infections in the semen (Merhi *et al*., 2018).

Since the use of busulfan as a cancer chemotherapy drug causes disturbances in the testicular spermatogenesis by oxidative stress, today these disorders are reduced by the use of antioxidants such as melatonin. Moreover, ozone therapy is used to treat some human diseases by stimulation of the endogenous antioxidant defense systems. In this study, we decided to investigate the effects of ozone therapy on the changes of serum concentration of testosterone, sperm parameters, MDA level, antioxidant enzyme activities, and total antioxidant capacity (TAC) level in male mice treated with busulfan. In the end, the results were compared with melatonin as a potent antioxidant agent.

## MATERIALS AND METHODS

We used twenty-four male mice aged 4-6 weeks and weighing 25 to 30 grams in this study. We kept the mice under the same environmental conditions (24±3 ºC, 12 hours of darkness and 12 hours of light), and fed them standard diet and water. A 7-day period was considered for the mice acclimatization with the environment (Ekici *et al*., 2011). The ethics committee on animal research at Ahvaz Jundishapur University of Medical Sciences approved the experimental protocol (IRAJUMSABHC.REC.1397.085).

We randomly divided the mice into four groups of six:

- Control group
- Busulfan group: Intraperitoneal busulfan injection (30 mg/kg) in one step (Mete *et al*., 2017)
- Busulfan group with melatonin: One-time busulfan injection followed by daily intraperitoneal melatonin (10 mg/kg) injection that continued for seven days (Ekici *et al.,* 2011)
- Busulfan group with ozone: One-time busulfan injection followed by daily intraperitoneal ozone (4 mg/kg) injection that continued for seven days (Ekici *et al*., 2011)

### Busulfan preparation

For the injection of busulfan (Sigma B2635, UK), the mice were first weighed and then proportional to their body weight, a small amount of busulfan powder was dissolved in dimethyl sulfoxide (DMSO). We injected the mice intraperitoneally with 0.1 ml of the prepared busulfan solution in proportion to the dosage used (Rohnavaz *et al*., 2016).

### Melatonin preparation

Melatonin (Sigma M5250) was first diluted in 1% ethanol and then injected intraperitoneally (Ekici *et al*., 2011).

### Ozone preparation

Ozone was obtained using a high quality medical oxygen gas containing approximately 3% of ozone/oxygen mixture (Gardina Co.). The ozone concentration was measured using ultraviolet light at 254 nm. Based on body weight of the mice, ozone was dosed at 4 mg/kg and was injected to them (Ekici *et al*., 2011).

After the end of the breeding period (35 days), the mice were anesthetized with one injection of ketamine and xylazine, and their blood was collected for measurement of testosterone. In the next step, their testicles were removed and weighed. One testis was used to make tissue slides. Hematoxylin-eosin staining was then carried out for evaluation using Johnson scoring, morphometric studies of seminiferous tubules (epithelial thickness and diameter of the tubules). The other testis was homogenized and frozen for measurement of MDA, SOD, CAT, and TAC levels. The percentages of sperm parameters (number, motility, and normal morphology) were determined based on epididymis spermatozoa.

### Evaluation of Johnson’s score for seminiferous tubules

The Johnson method was used to study the quality of seminiferous tubules (Lewis-Johnes & Kerrigan, 1985). This method has a score of 1-10. Based on the following criteria, each section of the seminiferous tubules was scored:

10: Complete spermatogenesis, a large number of sperms, plus round and regular tubules.
9: There is a large number of sperms, but there is no round and regular lumen of tubules.
8: The number of sperms is very low.
7: There is no sperm, but a large amount of spermatids.
6: There are a small number of spermatids.
5: There is no sperm or spermatogenesis. There is a large number of primary spermatocytes.
4: A very small number of primary spermatocytes.
3: No primary spermatocyte. Only spermatogonia are seen.
2: There is no reproductive cells. Only Sertoli cells.
1: Neither germ cells nor Sertoli cells are seen, nor the tubes are atrophic.

### Quantitative evaluation of seminiferous tubules

To examine the parameters of the tubules, we used the Motic Images software program. From each animal, 20 seminiferous tubules were randomly selected in round or almost round sections under a light microscope (40 x magnifications). The tubules that were elliptical or cut-off were not studied. The tubules’ diameters were calculated from the basal membrane of one side of the tubule to the basal membrane of the other side. Two diameters were calculated perpendicularly, and then the average of the anchors in each tubule was calculated in terms of micrometers. Using the exact method described above, we calculated the mean thickness of the germinal epithelium (Mete *et al*., 2017).

### Sperm parameters determination

The mice epididymis was separated after 35 days and the sperms were removed. Then, we calculated the sperm parameters (number, motility, and normal morphology).

### Measuring serum testosterone levels

To determine the level of testosterone, we placed the collected blood in a refrigerator for 24 hours. The specimens were centrifuged at 2000 rpm for 20 minutes and serum separation was performed and prepared according to the guidelines provided with the purchased kit (Ideal tashkhis, Iran). We used a radioimmunoassay (RDG, Germany) to determine serum testosterone levels (Mohammad Ghasemi *et al*., 2010a; 2010b).

### Measuring MDA, SOD, CAT, and TAC

All the chemical of this section were purchased from ZellBio GmbH (Germany). The testicular tissue used for biochemical studies was frozen in liquid nitrogen and then stored at -80ºC, until the tests were carried out. The tissue was homogenized in 1 ml of a 0.9% NaCl solution in ice, and then the sample was centrifuged at 1500 rpm for 10 minutes at 4ºC, which was used for measuring MDA (CAT NO. ZB-MDA-96A), SOD (CAT NO. ZB-SOD-96A), CAT (CAT NO. ZB-CAT-96A), and TAC (CAT NO. ZB-TAK-96A) (Mohammad Ghasemi *et al*., 2010a; 2010b). To measure the activity of these enzymes, an adequate volume of the sample and the prepared solutions were mixed according to the kit, and the optical absorption of the sample tubes and the standard wavelengths were read by a spectrophotometer.

### Statistical Analysis

We ran the data analysis using a Statistical Package for the Social Sciences (SPSS version 22). We represented the results as mean ± SD and P-value. Differences among the groups were analyzed by the nonparametric Kruskal-Wallis one-way analysis of variance. We then used the Mann-Whitney U test as a post hoc test for multiple comparisons. The significance level of *p* value was ≤0.05.

## RESULTS

### MDA levels

Since ROS can react with all the cellular components including polyunsaturated fatty acids in the cell membrane, and such reactions lead to different cellular injuries, ranging from increased membrane permeability to cell lysis (Sharma *et al*.,2017). We measured the lipid peroxides derived from unsaturated fatty acids in the studied groups using the MDA assay.

**Figure 1:**
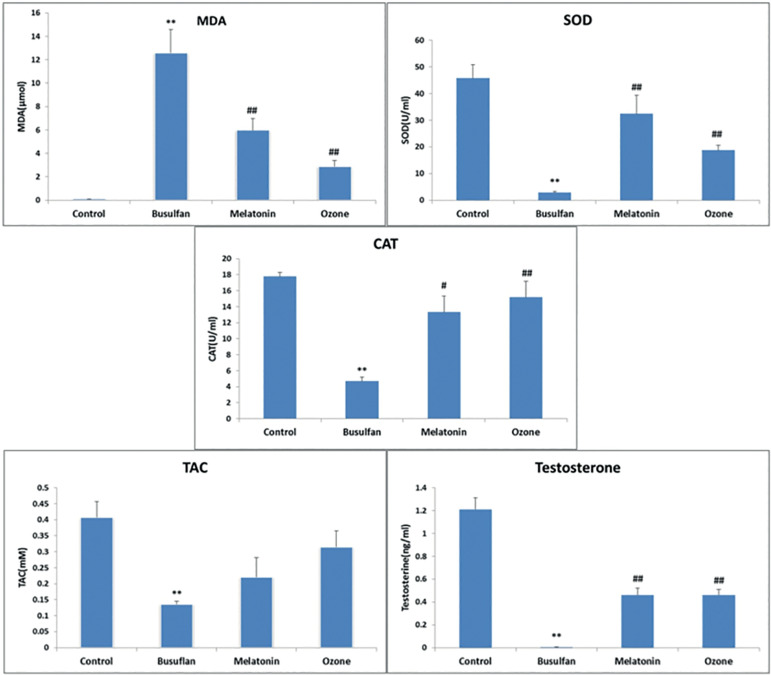
MDA, SOD, Catalase, TAC and testosterone biomarker levels in all study groups. Values are expressed as Means±SD. **p<0.01 or *p<0.05 compared with the control group, ##p<0.01 or #p<0.05 compared with the busulfan group.

The MDA level of testicular tissue in the busulfan group was significantly higher than the control group (*p*=0.007). Moreover, melatonin- and ozone-treated groups showed significant decreases of MDA levels versus the busulfan group (*p*=0.009). However, the effect of ozone therapy on MDA level was better than the melatonin, considering that this level in the ozone-treated group was similar to that of the control group (*p*=0.126) (Figure 1). These results showed that ozone could reduce the lipid peroxidation and permeability of cell membrane to ROS caused by busulfan.

In order to evaluate the antioxidant status of the testes, we evaluated SOD, CAT, and TAC levels in the all groups of this study.

### SOD levels

SOD is one of the antioxidant enzymes that scavengers ROS production in cells. It rapidly converts anion superoxide (O_2_) into low-risk hydrogen peroxide in cells and converts GPX and catalase into water (Peltola *et al*., 1992). In this study, busulfan caused a significant decrease in SOD compared to the control group (*p*=0.009). Melatonin and Ozone had good effects on SOD and increased this enzyme compared to the busulfan group (*p*=0.009); however, melatonin had a better therapeutic effect compared to the ozone group, and its results were similar to the control group (*p*=0.175) (Figure1).

### CAT levels

Another antioxidant enzyme for ROS scavengers in cells is catalase. The results of the present study showed that busulfan caused a significant reduction in tissue catalase compared to the control group (*p*=0.009). Moreover, melatonin and ozone were able to significantly increase the level of this enzyme compared to the busulfan group (melatonin-treated group: *p*=0.016 and ozone-treated group: *p*=0.009) and both of them reduced the effects of ROS caused by busulfan in the testes. In addition, the effects of melatonin and ozone on the catalase level were compared to the control group and the results were similar to it (melatonin-treated group: *p*=0.465 and ozone treated-group: *p*=0.076) and they had positive effects on the busulfan toxicity (Figure 1).

### TAC levels

TAC is a nonenzymatic antioxidant capacity and it was evaluated in this study. Busulfan caused a significant reduction in TAC level compared to the control group (*p*=0.008). Although in the two groups treated with ozone (*p*=0.242) and melatonin (*p*=0.517), TAC levels were increased compared to the busulfan group, but they did not have a significant effect on them and these substances could not reduce ROS by the TAC method (Figure 1).

Testosterone and sperm analyses were performed in this study to evaluate testis function.

### Testosterone levels

Testosterone in the group treated with busulfan showed a significant decrease compared to the control group (*p*=0.005). Blood testosterone was increased in both groups of ozone (*p*=0.004) and melatonin (*p*=0.004) compared to the busulfan group, and the results were similar to those of the control group (*p*=0.106 and *p*=0.067) (Figure 1). Moreover, they had a good effect on the busulfan toxicity.

### Sperm Analysis

Sperm analysis including count, motility, viability, and morphological characteristics had a significant difference in the busulfan group compared to the control group. In addition, there were significant changes in the ozone and melatonin groups compared to the busulfan group, which is shown in table 1.

**Table 1:** Count, viability, morphology and motility of control and experimental groups

Parameter	Count (×106/Ml)	viability (%)	morphology (%)	total motility (%)
Group				
Control	64.15±10.86	67.80±2.94	68.20±4.32	61.29±1.35
Busulfan	0.260±0.0894 p*=0.008	0±0 p*=0.005	0±0 p*=0.005	0±0 p*=0.005
Melatonin	50.10±1.431 *p*#=0.008	54.99±0.787 *p*#=0.005	45.40±2.60 *p*#=0.005	47.60±.5 *p*#=0.005
Ozone	52.30±4.29 *p*#=0.008	52.14±1.45 *p*#=0.005	48±4 *p*#=0.005	51.20±5.01 *p*#=0.005

Values are expressed as Means±SD.

* sign indicates the comparison of the studied groups with the control group

# sign indicates the comparison of the studied groups with the busulfan group.

To evaluate testes morphology in this study, we assessed their weight, seminiferous tubule diameters, of seminiferous tubules’ epithelium thickness, and Johnson’s score.

### Total testes weight

The total testicular weight in the busulfan group showed a significant decrease compared to the control group (*p*=0.009). In the ozone (*p*=0.009) and melatonin-treated groups (*p*=0.008), the testicular weight was significantly increased compared to the busulfan group (Table 2).

**Table 2:** Testis weight, tubule diameters, epithelium thickness and Johnson's score of control and experimental groups

Parameter	Control	Busulfan	Melatonin	Ozone
Group				
testis weight (g)	91.90±10.60	21.20±0.836 p*=0.009	33.60±10.03 *p*#=0.008	38±4.94 *p*#=0.009
Tubule diameters(µm)	139.74±7.211	100.7±19.34 p*=0.016	104.7±17.52 *p*#=0.597	132.78±7.84 *p*#=0.047
Epithelium thickness (µm)	103.28±3.51	54.76±23.25 p*=0.009	65.2±18.30 *p*#=0.115	97.2±8.09 *p*#=0.076
Johnson score	9.54±0.089	1.38±0.376 p*=0.008	6.62±0.349 *p*#=0.009	8.44±0.089 *p*#=0.008

Values are expressed as Means±SD.

* sign indicates the comparison of the studied groups with the control group and

# sign indicates the comparison of the studied groups with the busulfan group.

### Seminiferous tubules’ diameters

The diameters of the tubules in the busulfan group were significantly reduced compared to the control group (*p*=0.016), and while ozone was able to improve the seminiferous tubules (*p*=0.047), there were no significant changes in the melatonin-treat group in comparison to the busulfan group (*p*=0.597) (Table 2).

### Epithelium thickness of the seminiferous tubules

The seminiferous tubules’ epithelium thickness in the busulfan group was significantly reduced compared to the control group (*p*=0.009). Although ozone was able to improve the epithelium thickness, such changes were not statistically significant (*p*=0.076). Moreover, melatonin could not affect the epithelium thickness in terms of statistics (*p*=0.115) in comparison to the busulfan group (Table 2).

### Johnson’s score and testis histology

**Figure 2:**
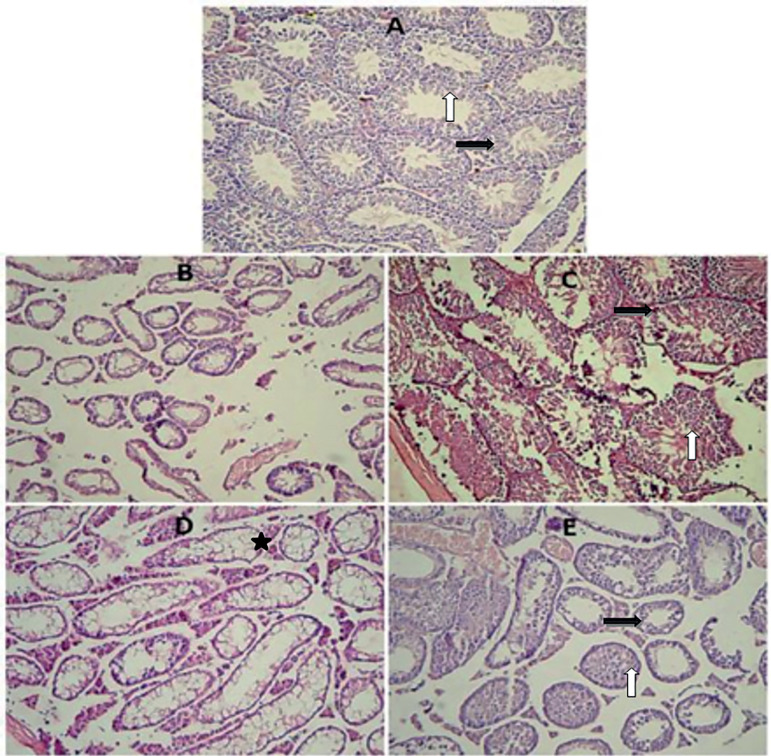
(A) A section from control group showing normal histological findings of preserved spermatogenesis. (B) This section of testis is from the busulfan group, showing damaged seminiferous tubules and interstitial tissue. (C) A section from the melatonin group showing preserved spermatogenesis up to the level of spermatocyte and Leydig cells. (D) A section from the melatonin group showing damaged and vacuolated tubules. (F) A section from the ozone group showing normal seminiferous tubules and interstitial tissue (white Arrows are showing spermatogenesis; black arrows are showing interstitial tissue with Leydig cells and Stars are showing vacuolated tubules. Hematoxylin and Eosin stain ×20.

The control group showed evidence of regular seminiferous tubular morphology. These tubules had normal spermatogenesis and interstitial tissue with Leydig cells. There was a significant decrease in the busulfan group in terms of Johnson’s score compared to the control group (*p*=0.008). In this group, the injury of seminiferous tubules did not have spermatogenesis, and the Leydig cells were not seen in the interstitial tissue. In this study, melatonin (*p*=0.009) and ozone (*p*=0.008) significantly improved the Johnson’s score compared to the busulfan group. Both melatonin- and ozone-treated groups had normal tubules with spermatogenesis and Leydig cells in the interstitial tissue. However, some tubules in the melatonin-treated group were vacuolated and did not have spermatogenesis or Leydig cells. The testes in the ozone-treated group also had distortion in their seminiferous tubular morphology, but the damage was less than that in the melatonin group (Figure 2) (Table 2).

## DISCUSSION

Busulfan is a potent agent that preferentially kills spermatogonial stem cells. In a clinical setting, an individual undergoing chemotherapy treatment often produces prolonged and sometimes irreversible depression of sperm count that affects male fertility. Today, researchers are looking for ways to eliminate the effects of busulfan toxicity in people who have cancer. One of these ways is the use of substances such as melatonin and antioxidants. Since ozone is currently used to treat several diseases, its effects were investigated in reducing the consequences of busulfan toxicity.

One of the effects of busulfan toxicity is disruption in the equilibrium of oxidative stress in cells. Oxidative stress is the result of an imbalance between ROS formation and antioxidants. Several defense systems are involved within the cells to prevent uncontrolled ROS increase. These systems include non-enzymatic molecules (glutathione, vitamins A, C, and E, and several antioxidants present in food) as well as enzymatic scavengers of ROS including superoxide dismutase, catalase, and glutathione peroxidase. Busulfan could conjugate with glutathione (Hassan *et al*., 2002) and NADPH (Probin *et al*., 2007), thus increasing oxidative stress and ROS. ROS react with any biochemical component of cells, proteins, DNA, and particularly lipids in the cell membrane and they can cause cell injury. Therefore, as demonstrated herein, busulfan could induce malondialdehyde (MDA) production and alter membrane fluidity and cell permeability, resulting in damaged cell structure and function (Cai *et al*., 2016). In addition, busulfan reduced enzymatic scavengers like SOD, CAT, and nonenzymatic antioxidant capacity (TAC) levels of testes, and it damaged and eliminated most of the spermatogonial cells (Smith *et al*., 2012).

In order to remove the adverse effects of busulfan on the testes, two substances of melatonin and ozone were used in this study. We demonstrated that similar to melatonin, ozone reduced MDA and increased SOD and CAT levels, and it reduced the effects of the ROS produced by busulfan through activating the antioxidant system. In agreement with the current study, Buyuklu *et al*. (2017) showed that ozone therapy, as an antioxidant, increased SOD and CAT levels, and decreased MDA levels on the cardiac function. In addition, a study (Tusat *et al*., 2017) on rats investigated the effects of ozone therapy on testicular damage due to ischemia/reperfusion, by assessing biochemical parameters such as Ischemia-Modified Albumin (IMA), Total Antioxidant Status (TAS), Total Oxidant Status (TOS), and Oxidative Stress Index. These findings suggest that ozone therapy is able to reduce the oxidative stress markers that usually rise in testicular torsion. In addition, a study showed that the mild oxidative stress caused by ozone leads to activation of antioxidant cascades that in turn, leads to the production of SOD, CAT, and glutathione S-transferase reaction (Inal *et al*., 2011). Another study revealed that the therapeutic efficacy of ozone therapy may be partially due the controlled and moderate oxidative stress produced by the reaction of ozone with several biological components such as transcription of antioxidant response elements (ARE). Transcription of ARE causes the production of numerous antioxidant enzymes in cells such as SOD, GPx, glutathione-s-transferase (GSTr), and catalase (CAT), and activates the antioxidant system in cells (Sagai & Bocci, 2011).

However, in this study, the effect of ozone was better than that of melatonin on MDA, and melatonin was better than ozone in terms of SOD level, while they had similar effects in their rates of catalase level. On the other hand, none of them had any effect on TAC level, which is similar to the results of Tusat’s study (Tusat *et al*., 2017). Therefore, this study showed that melatonin and ozone affect the enzymatic scavengers of ROS in different ways.

Another type of busulfan toxicity is damaging the gonadal organ that causes loss of spermatogenesis and fertility (Trevor *et al*., 2002). This study showed that busulfan could reduce testicular weight and spermatogenesis and, in these conditions, the testes histology was abnormal, as they did not have normal seminiferous tubules. In addition, the tubules had only Sertoli cells and a few spermatogonia cells. Moreover, the epithelium thickness, the seminiferous tubules diameter, and the Johnson’s score were significantly reduced. The mice used in this study had a few immotile and abnormal spermatozoa, and their testosterone levels were very low. This result is consistent with those from previous studies. Choi *et al*. (2004) showed that busulfan, by inhibiting cell proliferation, has the ability to produce apoptosis in testicular germ cells. Nasimi *et al.* (2016) explained in a study that busulfan led to a significant reduction in sperm parameters (number, motility, and morphology) and increased the wall destruction of seminiferous tubules. In another study, they showed that busulfan partially eliminates stem cells because of its alkylating nature (Ray, 2012), and it kills cells by producing free radicals (Ray, 2011). Therefore, it seems that busulfan inhibits the spermatogenesis process, especially by oxidative damage.

In this study, ozone therapy significantly reduced the effects of busulfan on sperm parameters, testosterone level, weight, testes histology, and seminiferous tubules’ diameter. In one study, Salem *et al*. (2017) evaluated the cytoprotective effects of rutin, ozone, and their combination on Adriamycin (ADR)-induced testicular toxicity in rats. They showed that treatment with rutin and/or ozone, improved sperm functions, testosterone level, luteinizing hormone, follicle stimulating hormone, testicular enzymes, and oxidant/antioxidant status parameters. They stated that ozone therapy alone, almost completely reversed the toxic effects of ADR and restored all parameters to normal levels. Moreover, Ekici et al. in 2012 indicated that ozone therapy improved the testicular rotation with histopathological parameters. They concluded that ozone was better than melatonin, and its effectiveness was comparable to it. In another study, Mete *et al*. (2017) concluded that ozone can improve the histopathological changes caused by the torsion. It has also been suggested that intratesticular ozone therapy is more effective than intraperitoneal therapy. Another study showed that cryptorchidism significantly reduced testicular atrophy index and sperm motility, while ozone treatment significantly recovered these parameters and Johnson’s scoring in the intervention groups (Biçer *et al*., 2018).

In addition, in this study, melatonin significantly reduced the effects of busulfan on sperm parameters and the testosterone level, as well as testes weight and histology. These results were consistent with other studies. Ferdosi Khosroshahi *et al*. (2013) showed that melatonin significantly increased the maintenance of sperm parameters, but it did not improve infertility. In a 2010 study, Mohammad Ghassemi et al. concluded that melatonin has protective effects on busulfan-induced injury in mice testes. Although they stated that the mechanism of this effect is not completely understood, it is likely done by reducing the oxidative damage. In another study, melatonin did not prevent the ischemia/reperfusion (IR)-induced reduction in sperm concentration. However, melatonin significantly decreased the sperm abnormalities and improved sperm morphology when compared with the IR-injured samples (Kurcer *et al.,* 2010).

However, treatment with ozone or melatonin improved the aforementioned parameters while ozone had better effects on Johnson’s score, testis histology, and seminiferous tubules’ diameters. This should be mentioned that these substances had no effect on the epithelium thickness of seminiferous tubules in this study. Because these substances have an acceptable effect on other parameters such as testosterone level and concentration and morphology of sperm, perhaps if the tests that were performed on mice were continued for a longer period after injection of the substances (more than 35 days), the thickness of tubular epithelium would be improved as well.

## CONCLUSION

This study showed that similar to melatonin, ozone can improve the effects of busulfan injury on mouse testis, and like antioxidants, it can regulate the antioxidant defense system of cells. However, further studies are required to understand the precise mechanism of ozone function on cells and organs.
